# Detection of Vaccinia Virus in Dairy Cattle Serum Samples from 2009, Uruguay 

**DOI:** 10.3201/eid2212.160447

**Published:** 2016-12

**Authors:** Ana Paula Moreira Franco-Luiz, Danilo Bretas Oliveira, Alexandre Fagundes Pereira, Mirela Cristina Soares Gasparini, Cláudio Antônio Bonjardim, Paulo César Peregrino Ferreira, Giliane de Souza Trindade, Rodrigo Puentes, Agustin Furtado, Jônatas Santos Abrahão, Erna Geessien Kroon

**Affiliations:** Universidade Federal de Minas Gerais, Belo Horizonte, Brazil (A.P.M. Franco-Luiz, D.B. Oliveira, A. Fagundes Pereira, M.C.S. Gasparini, C.A. Bonjardim, P.C.P. Ferreira, G. de Souza Trindade, J. Santos Abrahão, E.G. Kroon);; Universidade Federal dos Vales do Jequitinhonha e Mucuri, Diamantina, Brazil (D.B. Oliveira);; Universidad de la Republica Oriental del Uruguay Facultad de Veterinaria, Montevideo, Uruguay (R. Puentes, A. Furtado)

**Keywords:** vaccinia virus, orthopoxvirus, bovine, dairy cattle, poxvirus, Uruguay, zoonoses, buffalopox virus, South America, Uruguay, cattle, bovine vaccinia, viruses

## Abstract

We detected orthopoxvirus in 28 of 125 serum samples collected during 2009 from cattle in Uruguay. Two samples were PCR-positive for vaccinia virus and had sequences similar to those for vaccinia virus associated with outbreaks in Brazil. Autochthonous circulation of vaccinia virus in Uruguay and other South American countries cannot be ruled out.

Orthopoxviruses (family *Poxviridae*, genus *Orthopoxvirus*) cause several zoonotic diseases worldwide, including diseases caused by monkeypox virus in Africa, cowpox virus mainly in Europe, and vaccinia virus (VACV) in South America and Asia (1). During the past few decades, reports about emerging and reemerging zoonotic VACV and buffalopox virus have increased, as have the number of severe cases of disease caused by the viruses (2,3). VACV has been isolated in Brazil and detected in Argentina (*4**–**6*). During bovine vaccinia outbreaks, VACV affects mainly dairy herds; lesions develop on the animals, especially on the teats and udders, resulting in reduced milk production (*5**,**6*). In humans, most VACV infections occur in persons who milk cattle; infection frequently causes lesions on hands and forearms, but systemic clinical manifestations have been described and represent a challenge to public health services (*5*).

The first notifications of VACV detection in Brazil were in the 1960s and 1970s during a government surveillance campaign that investigated emerging pathogens in wild animals (*5*). However, it was not until 1999 that the first outbreaks of bovine vaccinia were reported in Brazil, when cases occurred in Rio de Janeiro and São Paulo States (*5*). Over the next few years, VACV spread to several more states; since then, all geographic regions of Brazil have been affected by bovine vaccinia, including states bordering other countries in South America, which explains the recent detection of VACV in Argentina (Figure 1) (*4**–**8*). Given these detections, serious concern exists regarding the potential spread of VACV to other countries in South America. Uruguay, a country that borders Brazil, has had no reports of VACV detection. To determine if the virus has spread to Uruguay, we investigated the presence of orthopoxvirus neutralizing antibodies and viral DNA in serum samples from cattle in the country.

**Figure 1:**
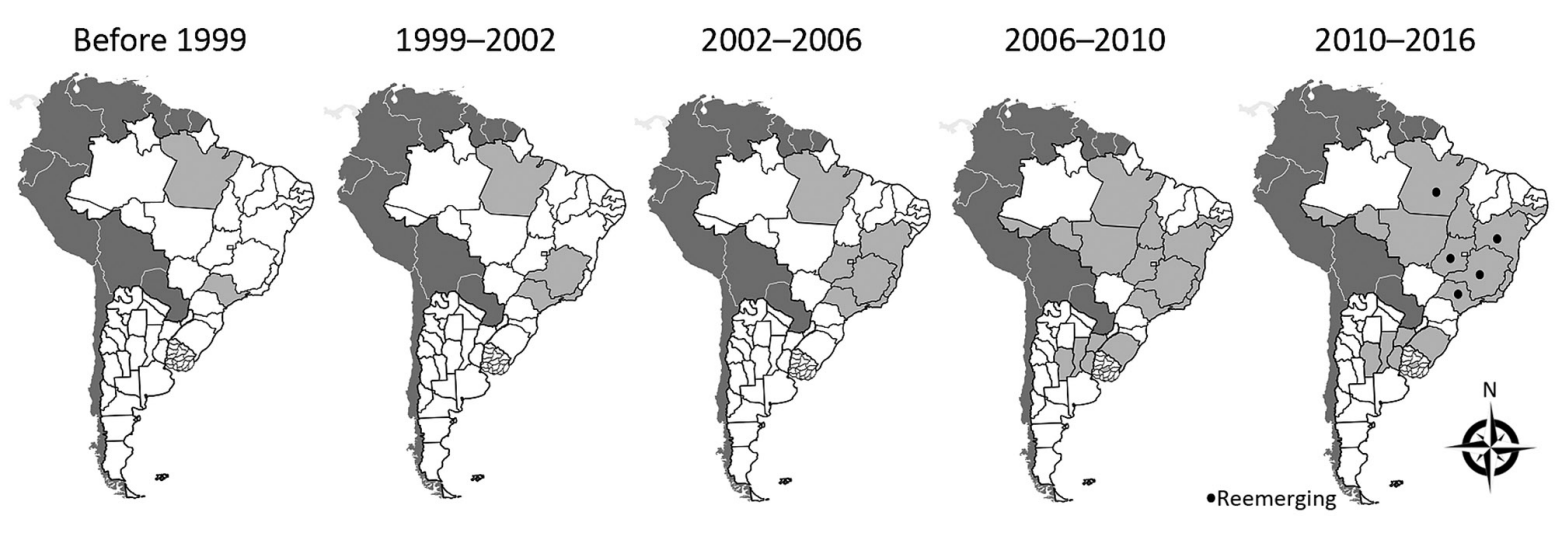
Chronologic representation of vaccinia virus (VACV) emergence and reemergence in South America. Dark gray indicates countries in which VACV outbreaks have not been officially described; light gray indicates states in Brazil, Argentina, and Uruguay where VACV outbreaks were detected by serologic or molecular testing; white indicates states in Brazil and Argentina where VACV has not been detected; black dots indicate areas where VACV is reemerging.

## The Study

**Figure 2:**
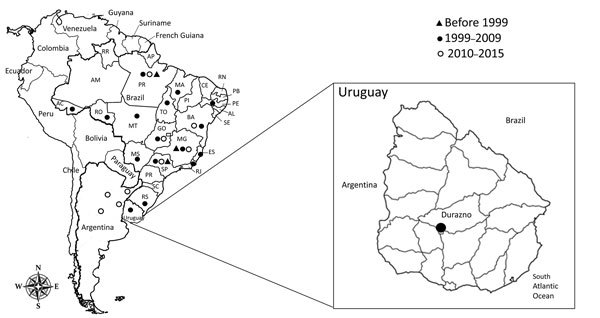
Chronologic detection of vaccinia virus in South America. Zoomed-in map shows location of Durazno County, Uruguay, where serum samples were collected from dairy cattle in 2009 to test for the presence of vaccinia virus. Brazil states: AC, Acre; AM, Amazonas; AL, Alagoas; AP, Amapá; BA, Bahia; CE, Ceará; ES, Espírito Santo; GO, Goiás; MA: Maranhão; MG, Minas Gerais; MS, Mato Grosso do Sul; MT, Mato Grosso; PA, Para; PB, Paraíba; PE, Pernambuco; PI, Piauí; PR, Paraná; RJ, Rio de Janeiro; RN, Rio Grande do Norte; RO, Rondônia; RR, Roraima; RS, Rio Grande do Sul; SC, Santa Catarina; SE, Sergipe; SP, São Paulo; TO, Tocantins.

We analyzed serum samples that were collected in May 2009 from 125 dairy cows in Durazno County (33°23′0′′S, 56°31′0′′W), Durazno State, Uruguay (Figure 2). The cattle herds had no clinical sings of disease at the time of serum collection. To determine the presence of neutralizing antibodies in the serum samples, we used an orthopoxvirus plaque-reduction neutralization test as previously described (*9*). The serum titer was defined as the highest dilution that inhibited >70% of virus plaques compared with negative controls (*4*).

Because previous studies have detected viral DNA in the serum of animals and humans with and without clinical manifestations (*4**,**10**,**11*), we performed a molecular investigation to identify orthopoxvirus. We used quantitative PCR (qPCR) to amplify VACV growth factor gene (C11R) DNA. This qPCR tool has high sensitivity and specificity and, thus, has been routinely used as an orthopoxvirus diagnostic tool by our group (*12*). For molecular characterization, we used the nonconserved orthopoxvirus hemagglutinin gene (A56R) (*13*). We used VACV–Western Reserve as the PCR-positive control for amplification and characterization.

The PCR A56R products obtained from C11R PCR–positive samples were sequenced in both orientations and subjected to capillary electrophoresis (3130 Genetic Analyzer, BigDye Terminator Cycle Sequencing Kit v3.1; Applied Biosystems, Foster City, CA, USA). We used the ClustalW (http://www.clustal.org/) method to align sequences with previously published orthopoxvirus sequences from GenBank; alignments were manually checked with MEGA6 (http://www.megasoftware.net/). We constructed phylogenetic trees using the neighbor-joining method with 1,000 bootstrap replicates and the Tamura 3-parameter model in MEGA6. All field and laboratory clinical samples were processed separately to avoid cross-contamination. Serologic and molecular tests were performed in 2 independent experiments and in duplicate.

We detected neutralizing antibodies against orthopoxvirus in 28 (22.4%) of 125 serum samples from cattle in Uruguay; titers were 100 neutralizing units (NU)/mL in 10 (35.7%) samples, 200 NU/mL in 11 (39.3%) samples, 400 NU/mL in 5 (17.9%) samples, and 800 NU/mL in 2 (7.1%) samples (Technical Appendix Figure 1, panel A). To confirm that the orthopoxvirus seropositivity represented seropositivity to VACV, we used the C11R gene to molecularly test DNA in the serum samples; 2 (1.6%) samples were positive by qPCR, and 1 of those was also positive by the plaque-reduction neutralization test. 

One of the 2 C11R PCR–positive isolates was also positive for the A56R gene; we sequenced the gene and named the strain VACV Uruguay. Alignment of the A56R nucleotide sequence showed the presence of an 18-nt signature deletion, which is also present in sequences of Brazilian-VACV group I, but not group II, viruses. Unlike sequences for other VACVs, the sequence for VACV Uruguay had an A→T polymorphism (Technical Appendix Figure 2, panel A). VACV Uruguay exhibited higher identity with group I (98.6% identity) viruses from Brazil and Argentina than to group II (97.3% identity) viruses from Brazil (Technical Appendix Figure 1, panel B). Furthermore, in the phylogenetic tree based on A56R nucleotide sequences, the VACVs from Uruguay clustered with group I VACVs that had been detected during outbreaks in Brazil and with viruses from Argentina (Technical Appendix Figure 2, panel B).

## Conclusions

Since 1999, VACV has been isolated from symptomatic and asymptomatic cattle, humans, and wildlife from the north to the extreme south of Brazil (*5**,**6**,**8*), and in 2014, VACV was described in bovine serum samples from Argentina (*4*). Although no exanthematous VACV outbreaks have been reported among cattle in Uruguay, we detected orthopoxvirus antibodies and VACV DNA in serum samples from dairy cattle in the country, indicating they have been exposed to VACV. Uruguay shares a border with Brazil, and its western border is shared with Entre Rios Province in Argentina, where VACV DNA has been detected. In addition, Uruguay shares its northern and eastern borders with Rio Grande do Sul State, Brazil, where Pelotas VACV has been isolated from horses (*4*,*5*). 

Our finding of orthopoxvirus antibodies and VACV DNA indicates a possible undetected or silent circulation of VACV in Uruguay. Considering the importance of the livestock sector in all countries of South America, concern exists about the possible spread of VACV beyond Brazil, Argentina, and Uruguay (*4**–**6**,**14*). Despite surveillance by veterinarians, efforts to stop the spread of VACV at borders may be hampered by the movement of infected rural workers, the marketing of asymptomatic live animals, and the misdiagnosis of VACV infection. Furthermore, VACV has been shown to circulate in wild environments, and it has been hypothesized that rodents may serve as VACV hosts, as they do for other orthopoxviruses, and facilitate the spread of VACV in border areas (*15*). 

Bovine vaccinia outbreaks in South America were first reported in Brazil, but we cannot rule out the possibility of autochthonous circulation of VACV in Uruguay and other countries in South America. Additional studies are needed to elucidate VACV seroprevalence in other countries in South America, and further research is needed to clarify the transmission pathways related to the spread of VACV in South America.

Technical Appendix Orthopoxvirus antibody titers in serum samples from 125 cattle in Durazno, Uruguay, and molecular characterization of a newly isolated virus, VACV Uruguay.
