# Supplementary material for: Detection of Vaccinia Virus in Dairy Cattle Serum Samples from 2009, Uruguay
Source: Emerg Infect Dis. 2016 Dec;22(12):2174–7. doi: 10.3201/eid2212.160447 (PMC5189138; doi:10.3201/eid2212.160447)
Supplement: Technical Appendix — Orthopoxvirus antibody titers in serum samples from 125 cattle in Durazno, Uruguay, and molecular characterization of a newly isolated virus, VACV Uruguay. [file 16-0447-Techapp-s1.pdf]

Article DOI: http://dx.doi.org/10.3201/eid2212.160447

Detection of Vaccinia Virus in Dairy Cattle Serum Samples from 2009, Uruguay

Technical Appendix

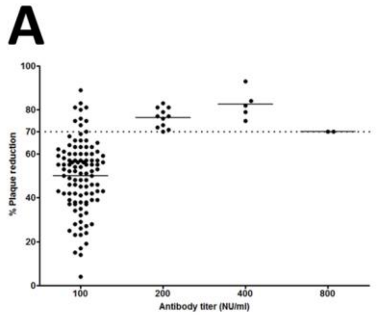

B

VACV_WR_(AY243312)	VACV WR	VACV BeAn	VACV Guarani P1	VACV Pelotas 1	VACV Belo Horizonte	**VACV Uruguay**	VACV Pelotas 2	VACV Serro	VACV Guarani P2	VACV Passatempo	VACV Aracatuba	VACV Argentina Corrientes	VACV Argentina Entre Rios	VARV Somalia	VARV Ehiopia	CPXV BR	MPXV Sierre leone	MPXV Zaire
VACV BeAn (DQ206442)	100,00																	
VACV Guarani P1 (DQ206436)	100,00	100,00																
VACV Pelotas 1 (GU183769)	100,00	100,00	100,00															
VACV Belo Horizonte (DQ206435)	100,00	100,00	100,00	100,00														
**VACV Uruguay**	97,26	97,26	97,26	97,26	97,26													
VACV Pelotas 2 (GU183770)	98,63	98,63	98,63	98,63	98,63	98,63												
VACV Serro (EF063677)	98,63	98,63	98,63	98,63	98,63	98,63	100,00											
VACV Guarani P2 (DQ206437)	98,63	98,63	98,63	98,63	98,63	98,63	100,00	100,00										
VACV Passatempo (DQ070848)	98,63	98,63	98,63	98,63	98,63	98,63	100,00	100,00	100,00									
VACV Araçatuba (AY523994)	98,63	98,63	98,63	98,63	98,63	98,63	100,00	100,00	100,00	100,00								
VACV Argentina Corrientes	98,63	98,63	98,63	98,63	98,63	98,63	100,00	100,00	100,00	100,00	100,00							
VACV Argentina Entre Rios	98,63	98,63	98,63	98,63	98,63	98,63	100,00	100,00	100,00	100,00	100,00	100,00						
VARV Somália (DQ441438)	68,49	68,49	68,49	68,49	68,49	65,75	67,12	67,12	67,12	67,12	67,12	67,12	67,12					
VARV Etiópia (DQ441424.)	68,49	68,49	68,49	68,49	68,49	65,75	67,12	67,12	67,12	67,12	67,12	67,12	67,12	100,00				
CPXV BR (AF482758)	72,60	72,60	72,60	72,60	72,60	69,86	71,23	71,23	71,23	71,23	71,23	71,23	71,23	95,89	95,89			
MPXV Sierre Leone (AY741551)	97,26	97,26	97,26	97,26	97,26	94,52	95,89	95,89	95,89	95,89	95,89	95,89	95,89	67,12	67,12	71,23		
MPXV Zaire (AF380138)	97,26	97,26	97,26	97,26	97,26	94,52	95,89	95,89	95,89	95,89	95,89	95,89	95,89	67,12	67,12	71,23	100,00	

Technical Appendix Figure 1. A) Antibody titers for virus neutralization in 125 cattle serum samples from Durazno, Uruguay. The samples were considered positive if they reduced at least 70% of the virus plaques. B) A56R (HA) gene nucleotide identity matrix. The analysis involved 18 nt sequences. Evolutionary analyses were conducted in MEGA 6.

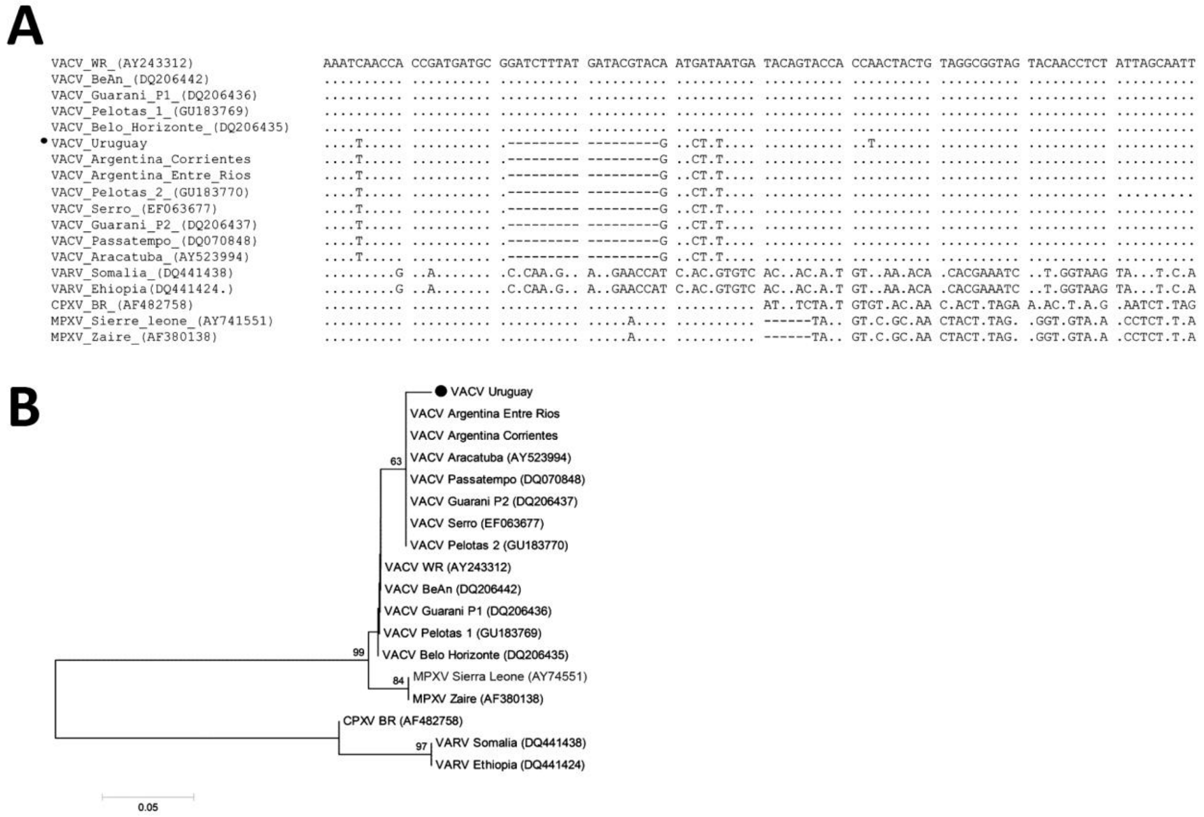

Technical Appendix Figure 2. A) Nucleotide sequence of the vaccinia virus (VACV) Uruguay A56R hemagglutinin (HA) gene (indicated by dot) compared with homologous sequences from several other *Orthopoxviruses*. VACV Uruguay showed one polymorphism (A→T) in comparison with other VACVs. B) Phylogenetic tree based on nucleotide sequences of orthopoxvirus HA gene (indicated by dot). The tree was constructed with HA sequences by using the neighbor joining method with 1,000 bootstrap replicates and the Tamura 3-parameter model in MEGA6 (http://megasoftware.net/). Bootstrap values are represented on branches; only values >60% are shown. Nucleotide sequences were obtained from GenBank. Scale bar represents 0.05 substitutions per nucleotide position.
